# Chitosan Modified Zeolite Molecular Sieve Particles as a Filter for Ammonium Nitrogen Removal from Water

**DOI:** 10.3390/ijms21072383

**Published:** 2020-03-30

**Authors:** Yunan Gao, Jiayu Zhang

**Affiliations:** 1 School of Municipal and Environmental Engineering, Shenyang Jianzhu University, Shenyang 110168, China; zhangjiayu9611@163.com; 2 Institute for Frontier Materials, Deakin University Geelong, Waurn Ponds, VIC 3216, Australia

**Keywords:** chitosan, zeolite molecular sieves, ammonium nitrogen, filtration

## Abstract

Drinking water containing a high amount of ammonium-nitrogen (NH_4_^+^-N) is not effectively removed by conventional treatment processes and can cause eutrophication. In this research, a composite adsorbent based on chitosan crosslink with zeolite molecular sieve (CTS-ZMS) was prepared for NH_4_^+^-N removal through dynamic adsorption filter experiments. Effect of bed depth (30, 50 and 70 cm), flow rate (32, 49 and 65 mL/min), initial pH value (4.5, 6.5 and 8.5) and influent NH_4_^+^-N concentration (3, 5 and 7 mg/L) was examined by using a filter column packed with CTS-ZMS particles. The Thomas model was applied to study the breakthrough curves and adsorption capacity. The optimal process parameters of the aforementioned factors were obtained at bed depth of 70 cm, flow rate of 32 mL/min, pH of 6.5 and initial NH_4_^+^-N concentration of 7 mg/L. Scanning electron microscopy (SEM), energy dispersive X-ray spectroscopy (EDS) and Fourier Transform Infrared Spectroscopy (FTIR) were investigated to analyze the structure and morphology of the CTS-ZMS adsorbents before and after 3 months running. The EDS and FTIR results showed Na^+^ and the active functional groups of -OH, -NH_2_ and -COO^−^ on CTS-ZMS adsorbent particles reacted with ammonium nitrogen. The results of this study supported the use of CTS-ZMS to improve drinking water filtration processes by increasing ammonium nitrogen reductions.

## 1. Introduction

Drinking water sources of most cities and villages in China are affected by ammonium nitrogen pollution [1]. Due to the rapid development of industrial and municipal activities, the excessive accumulation of ammonium nitrogen in industrial wastewater and domestic sewage are disposed into surface water and ground water [2,3]. The concentration of ammonium nitrogen from different sources is very high. For example, the ammonium nitrogen concentration in industrial-based wastewater may be in the range of 5–1000 mg/L and in the municipal wastewater is the range of 10–200 mg/L [4]. Such high concentration of ammonium nitrogen in water can cause serious environmental problems, like the depletion of dissolved oxygen, accelerated eutrophication of lakes and rivers and toxicity in fish and other aquatic animals [5,6]. In water solution, according to the existing pH, the ammonia (NH_3_) is usually found in the form of NH_4_^+^-N. This fact approximates the concentration of all reduced nitrogen in the form of ammonia and combined ammonium, which is known as ammonium nitrogen [7]. The most commonly used treatment options for ammonium nitrogen in drinking water are breakpoint chlorination and biological treatment, such as nitrification in filters [8]. However, if the ammonium nitrogen concentration in source water presents high and unstable data, a higher dose of chlorine is needed during the disinfection process to reach the breakpoint chlorination, thus high levels of toxic disinfection by-products (DBPs) will form [9]. NH_4_^+^-N can be consumed by nitrifying bacteria to form nitrate and nitrite in biological treatment process, and high nitrite will pose an acute health hazard [10]. In addition, biological activity of bacteria is greatly affected by water temperature and ammonium nitrogen concentration, which will affect the efficiency of biological reaction process [11]. Thus, less-cost, less-DBP and less environmental impact methods for ammonium nitrogen removal from polluted water are needed.

Adsorption is the most commonly used method of drinking water treatment process because of its selectivity, low cost and high availability [12]. Sorption with polysaccharides-based materials (such as chitosan [13] and alginate [14,15]) may be used for the removal of a big amount of organic and inorganic pollutants (the process is cost-efficient), and this can be used in conjunction with several techniques (such as flocculation-coagulation and filtration). Chitosan (CTS) has a wide application prospect in the field of water treatment due to its characteristics of wide source, safe and nontoxic and being environment-friendly [16]. Chitosan molecular structure contains amino NH_2_−, hydroxyl OH− and other reactive functional groups, which can be a variety of chemical modification, and can be cross-linked with the filter media to form multi-functional composite materials [17]. These materials have the effect of removing ammonia pollutants. Bernardi et al. (2018) studied three commercial chitins and chitosans in the removal of total ammonia synthetic effluents with different initial concentrations of ammonia [18]. Zadinelo et al. (2015) also evaluated the ability of removing ammonia from three clays in natural aquaculture effluent [19]. Chung et al. (2005) tested three chitosan prepared from crab exoskeleton with different molecular weights and deacetylation grade in the removal of ammonia [20]. However, chitosan is very sensitive to pH, being soluble in acidic media [21]. In addition, chitosan powder has no fixed shape when it is used alone and is easy to be lost during the treatment process. Therefore, it is necessary to find a supporting particle that chitosan could attach on stably without loss and resistance to acidic environment [22]. 

Zeolite molecular sieve is a synthetic aluminosilicate with an Si-Al-Si framework tetrahedral structure. Due to its regular internal pores, large specific surface area and stable structure, it has more efficiency adsorption performance than natural zeolite, and often is used as a carrier for adsorption separation [23]. Teimouri et al. (2016) prepared chitosan/zeolite Y/nano ZrO_2_ composite for the removal of nitrate from water [23]. Dehghani et al. (2017) investigated chitosan/zeolite composite for methylene blue dye removal from aqueous solutions [24]. However, these researchers have investigated chitosan/zeolite composite for water treatment in statistical adsorption experiments, the optimal adsorption parameters and adsorption capacities results by statistical tests are not suitable for a filtration application [25]. Compared with static adsorption, the dynamic adsorption used a packed column system and will be a better approach in actual water treatment processes. Dynamic adsorption experiments on the removal of organic micropollutants by using cross-linked chitosan/zeolite has been reported recently [26]. However, there are few literatures on the dynamic adsorption of ammonium nitrogen. 

In this study, dynamic adsorption for ammonium nitrogen removal from water by chitosan/zeolite molecular sieve (CTS-ZMS) fixed-bed column was investigated. The effects of bed depth, flow rate, the initial pH value and influent ammonium nitrogen concentration were assessed for optimizing the operation conditions. The Thomas model was used to analyze the dynamic adsorption. The surface physicochemical properties of CTS-ZMS after long term filtration were analyzed by scanning electron microscopy (SEM), energy dispersive X-ray spectroscopy (EDS) and Fourier Transform Infrared Spectroscopy (FTIR). This study provided a potential adsorbent for the development of a cheap and efficient cycle retained in ammonium nitrogen polluted water treatment.

## 2. Materials and Methods

### 2.1. Materials

Zeolite molecular sieve (NaA type, Brunauer–Emmett–Teller (BET) surface area 7.770 m^2^/g, pore volume 0.113 cm^3^/g, average pore diameter 4.403nm) was purchased from Shanghai New Molecular Sieve Co., Ltd. (Shanghai, China). NH_4_^+^-N was prepared with ammonium chloride (NH_4_Cl, 99.8%), Chitosan was 90% deacetylated, glutaraldehyde (25vol.%), potassium tartrate (0.8 vol.%), hydrochloric acid (HCl), acetic acid (CH_3_COOH), sodium hydroxide (NaOH), sodium carbonate (Na_2_CO_3_), sodium chloride (NaCl), calcium chloride (CaCl_2_) were purchased from Sinopharm Chemical Reagent Co., Ltd. All reagents used in the experiments were analytical reagent grade.

### 2.2. Preparation of Sorbent Media

CTS-ZMS was synthesized according to the method of previous research [27]. First, 7g of chitosan was added into a 250 mL flask containing 100 mL acetic acid solution (4 vol.%), the mixture was configured as chitosan acetate solution. Then, 100g of zeolite molecular sieve washed with deionized water and heated at T = 105 °C for 2 h were added to 100 mL glutaraldehyde (25vol.%) for 4 h. After mixing, the solid precipitates of zeolite molecular sieves with glutaraldehyde were added to chitosan acetate sol, and stirred at the speed of 130 rpm for 10 h at temperature of 30 °C to obtain the sorbent media, the mass ratio (m/m) of corresponding zeolite molecular sieve to chitosan was 100:7. Afterwards, the solid products were added to distilled water to remove acetic acid and glutaraldehyde, dried in the oven at 60 °C for 5 h and stored in a desiccator until use.

### 2.3. Batch Adsorption Experiment

Adsorption isotherm studies were carried out at NH_4_^+^-N concentrations of 2, 4, 6, 8, 10, 12 mg/L in contact with 100 mL of a 1.0 g CTS-ZMS adsorbent respectively during 8 h at 200 rpm and 25 °C temperature.

The adsorption process was commonly explained by the Langmuir (Equation (1)) [28] and the Freundlich (Equation (2)) [29] equations, which are listed below, respectively:

(1) $$\frac{C_{e}}{q_{e}}=\frac{C_{e}}{q_{m}}+\frac{1}{q_{m}K_{L}}$$

(2) $$q_{e}=K_{F}C_{e}{}^{1/n}$$

where *q_e_* is the adsorption capacity (mg/g) and *q_m_* (mg/g) indicates the maximum adsorption capacity. *K_L_* (L/mg) is Langmuir isotherm coefficient, 1/n is the Freundlich constant representing adsorption capacity and *K_F_* (mg/g) is the Freundlich constant representing adsorption intensity.

### 2.4. Dynamic Adsorption Experiment

**Figure 1:**
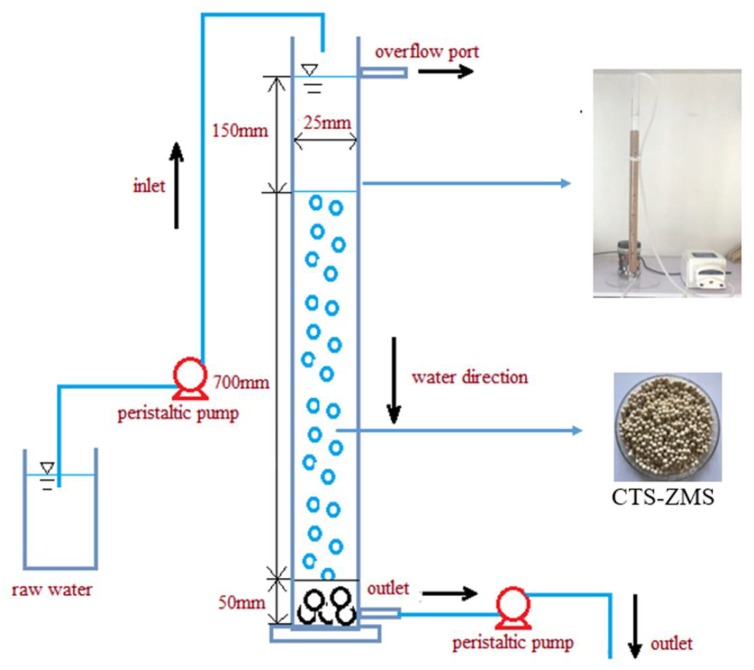
Schematic of chitosan crosslink with zeolite molecular sieve (CTS-ZMS) composite dynamic adsorption equipment.

A glass round column with 25 mm of internal diameter and 1000 mm of length was filled with CTS-ZMS particles for the adsorption experiment (Figure 1). To avoid the CTS-ZMS particles outflow from the column, a 50 mm height of cobblestone was used in the bottom of the column. Before the adsorption tests, distillated water was passed through the column for about 10 min to wash the particles and adjust the flow rate. Raw water polluted by ammonium nitrogen was prepared with ammonium chloride, the concentration of NH_4_^+^-N was simulated groundwater in Shenyang, China (average NH_4_^+^-N concentration: 3–7 mg/L). Raw water was down-flow transferred by a peristaltic pump at different flow rates. During the long term running of the filter, it was backwashed according to the effluent NH_4_^+^-N concentration. When the effluent NH_4_^+^-N concentration (*C_t_*) approached the inlet concentration (*C_0_*), i.e., *C_t_*/*C_0_* = 0.9, the column was backwashed by distillated water.

Optimal adsorption parameters of the CTS-ZMS in the column were studied by varying the bed depth (30, 50 and 70 cm), flow rates (32, 49 and 65 mL/min), influent pH value (4.5, 6.5, 8.5), influent ammonium nitrogen concentrations (3.0, 5.0 and 7.0 mg/L), respectively. The effluent solutions were collected every 30 min with a volume of 30 mL, to determine the residual NH_4_^+^-N concentration. The NH_4_^+^-N concentration was measured by a UV–vis spectrophotometer (752, Shanghai Spectrum Instrument Co., Ltd. China), using the standard methods provided by China Environmental Protection Industry Standard, including Water Quality-Determination of ammonium nitrogen Nessler’s reagent spectrophotometry (HJ 535-2009).

The adsorption performance of CTS-ZMS filter was evaluated by breakthrough curves. The dimensionless concentration, *C_t_/C_0_*, was plotted versus time. When the ammonium nitrogen effluent concentration is 10% (*C_t_/C_0_* = 0.1) and 90% (*C_t_/C_0_* = 0.9) of the influent concentration, the corresponding time is the breakthrough appearance time (*t_a_*) and exhaustion time (*t_b_*) [30].

### 2.5. Thomas Model

The Thomas model was used to evaluate the adsorption capacity of the CTS-ZMS filter and its breakthrough curves [31]. This model was expressed through the second-order law of kinetic reaction and the Langmuir isotherm without the presence of axial dispersion, even when the bed depth was at the minimum and the breakthrough occurred immediately after the flow started [32]. The Thomas model is applied to fit the dynamic adsorption data. The following equations are the mathematical expression (Equation (3)) and linearized form equation (Equation (4)) of Thomas model:

(3) $$\frac{C_{t}}{C_{0}}=\frac{1}{1+\exp\left(\frac{K_{Th}q_{0}m}{Q}-K_{Th}C_{0}t\right)}$$

(4) $$\ln\left(\frac{C_{0}}{C_{t}}-1\right)=\frac{K_{Th}q_{0}m}{Q}-K_{Th}C_{0}t$$

where *C_t_* (mg/L) is the effluent NH_4_^+^-N concentration and m (g) is the mass of adsorbent packed in the column. *C_o_* is the influent ammonium nitrogen concentration. *K_Th_* is Thomas coefficient (L/(mg min)), *q*_0_ stands for equilibrium adsorption after fitting the Thomas model (mg/g). *Q* is the volumetric flow rate (mL/min). A straight line can be obtained from a plot of ln(*C_0_/C_t_*− 1) against total flow time *t* (min) [33].

### 2.6. Characterizations

The characterizations of CTS-ZMS particles before and after filtration were analyzed by SEM, BET, EDS and FTIR. The morphology of CTS-ZMS was characterized by scanning electron microscopy (SEM, S4800 HITACHI, Japan), the Brunauer–Emmett–Teller (BET) surface area analysis was applied by using a Micromeritics ASAP2010 instrument to determine the specific surface area, and elemental mapping imaging was carried out with the energy dispersive X-ray spectroscopy (EDS, X-act Oxford, England). FTIR spectra were obtained on FTIR spectrophotometer (Tensor 27, Burker, Germany) with the scanning range from 4000 to 400 cm^−1^.

### 2.7. Regeneration

A total of 1.0 g of CTS-ZMS particles saturated with ammonium nitrogen was placed in 100 mL of 1 mol/L NaOH, 1 mol/L Na_2_CO_3_, 1 mol/L NaCl, 1 mol/L HCl and 1 mol/L CaCl_2_ solution, respectively. At 25 °C temperature, the rotation speed was 130 rpm, and the oscillation time was 24 h. Then, the adsorbed particles after regeneration of different chemical regenerants were rinsed with deionized water, and dried at a temperature of 60 °C for 5 h. Regeneration rate (Rr, %) was calculated as described in following Equation (5).

(5) $$R_{r}=\frac{q_{er}}{q_{en}}\times 100\%$$

where *Rr* is the regeneration rate(%). *q_er_* is the equilibrium adsorption capacity of regeneration CTS-ZMS particles, mg/g. *q_en_* is the equilibrium adsorption capacity of new CTS-ZMS particles, mg/g.

## 3. Results

### 3.1. Batch Experiment

**Figure 2:**
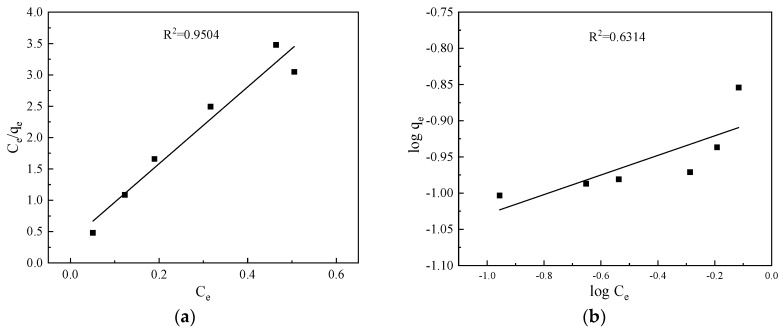
Adsorption isotherms for ammonium nitrogen adsorption: (**a**) Langmuir; (**b**) Freundlich.

Before the dynamic adsorption experiment, the isotherm adsorption tests were conducted to investigate the adsorption performance of CTS-ZMS adsorbents. The Langmuir and Freundlich isotherm model results were shown in Figure 2 and Table 1. Langmuir adsorption isotherm model (*R^2^* = 0.9504) can better describe the behavior of CTS-ZMS adsorption than Freundlich isotherm model (*R^2^* = 0.6314), indicating that the Langmuir adsorption isotherm model can more accurately describe the adsorption of ammonium nitrogen by CTS-ZMS. In the process, according to the Langmuir adsorption isotherm, the maximum adsorption capacity (*q_m_*) of CTS-ZMS to ammonia nitrogen was 1.145 mg/g. It was generally considered that when 0.1 < 1/n < 0.5, adsorption process was easy to proceed, and in the isothermal formula, 1/n = 0.137, so the adsorption reaction was easy to proceed [23].

Other literature studies on the removal of ammonium nitrogen by chitosan-based and zeolite-based adsorbents were listed in Table 2. Comparing the batch test results of chitosan [18], natural zeolite [34] and CTS-ZMS [27] for NH_4_^+^-N removal, the removal rates of each adsorbent were 29.74%, 69% and 81.6%, respectively, which showed the composite material of CTS-ZMS had better removal efficiency than the other two chitosan and natural zeolite uniquely material. Yang et al. (2014) investigated the NaA zeolite crosslinked with chitosan for ammonium nitrogen removal [35]. At the condition of dose 0.7 g/L, initial concentration 100 mg/L and temperature 25 °C, the adsorption capacity and removal efficiency were 5.84 mg/g and 82.8%, respectively. 

### 3.2. Dynamic Experiment

#### 3.2.1. Effect of Bed Depth

**Figure 3:**
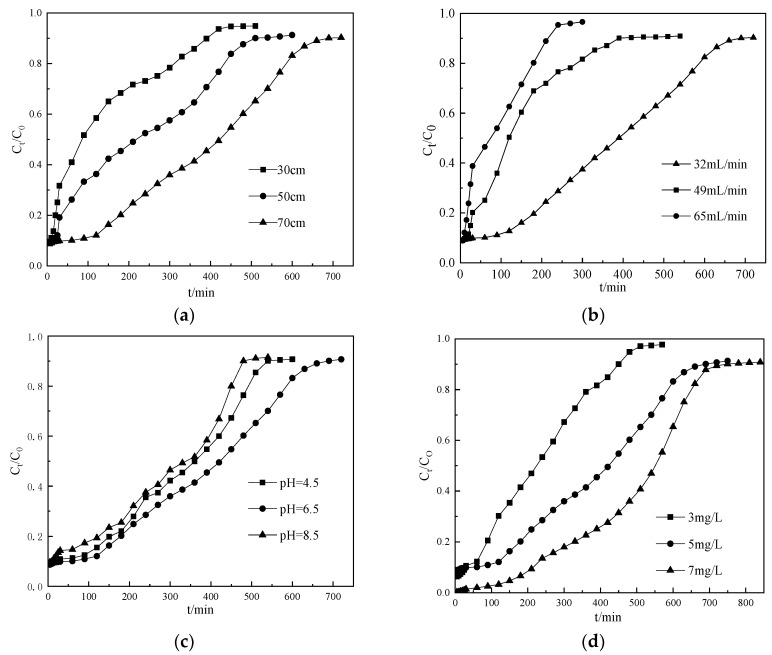
Breakthrough curves of ammonium nitrogen at different (**a**) bed depth, (**b**) flow rate, (**c**) influent pH, (**d**) initial ammonium nitrogen concentration from a fixed-bed column packed with CTS-ZMS.

Figure 3a showed the breakthrough curves of ammonium nitrogen at different bed depth (30 cm, 50 cm, 70 cm) from the CTS-ZMS fixed-bed column with the conditions of influent ammonium nitrogen concentration 5 mg/L, flow rate 32 mL/min, pH 6.5. The exhaustion time and adsorption capacity of ammonium nitrogen solution increased with increasing bed depth as expected. The adsorption capacity *q*_0_ at 30, 50 and 70 cm bed depth were evaluated as 0.225, 0.228 and 0.255 mg/g, respectively, as listed in Table 3. The weight of each column material at 30, 50 and 70 cm bed depth were 100, 170 and 240 g, respectively, therefore the column adsorption capacity of whole adsorbent packed in the column at 30, 50 and 70cm bed depth were evaluated as 23.28 mg/100 g, 39.16 mg/170 g and 61.20 mg/240 g, respectively. Since for a fixed initial ammonium nitrogen concentration (5 mg/L), increasing the bed depth provided more adsorbents and larger surface area, thus increased adsorption capacity; and decreasing the bed depth or the mass of CTS-ZMS led to lower adsorption capacity, faster breakthrough and column exhaustion [31,36]. Therefore, the bed depth greatly influenced the filter column breakthrough time (*t*) and the adsorption capacity (*q*_0_). The optimum bed depth was obtained approximately 70 cm which was corresponded to the mass of 240 g of the CTS-ZMS particles.

#### 3.2.2. Effect of Flow Rate

The effect of flow rate of the ammonium nitrogen solution on the CTS-ZMS dynamic filter column was investigated at the flow rates of 32, 49, 65 mL/min while maintaining the same bed depth of 70 cm, initial pH value of 6.5, influent ammonium nitrogen concentration of 5 mg/L. As shown in Figure 3b and Table 3, the breakthrough time of CTS-ZMS filter decreased with the increasing flow rate, the adsorption capacity *q*_0_ of the CTS-ZMS filter for ammonium nitrogen removal increased from 0.123 mg/g to 0.255 mg/g (Table 3) with the flow rate decreasing from 65 mL/min to 32 mL/min. The adsorption breakthrough time decreased at high flow rate and resulted in lower contact time between the ammonium nitrogen and adsorbents. In this study, optimal flow rate was chosen as 32 mL/min. 

#### 3.2.3. Effect of Initial pH Value

For drinking water treatment process, the pH value is an important factor that influences the adsorption capacity. The effect of pH value on the adsorption performance of ammonium nitrogen from water by the CTS-ZMS filter column was shown in Figure 3c and Table 3. The results showed that the breakthrough time increased with increasing pH within the range of 4.5–6.5, and then decreased in the range of 6.5–8.5. Therefore, the optimum and effective ammonium nitrogen adsorption happened at the pH value of 6.5. Table 3 listed the adsorption capacity *q*_0_ for pH of 4.5, 6.5 and 8.5 were measured as 0.215,0.255 and 0.190 mg/g, respectively. When the solution was an acidic environment (Ph = 4.5), the column adsorption capacity was lower than Ph = 6.5, which was attributed to the positively charged amine groups (-NH_2_) of chitosan that became protonated at acidic pH (-NH_3_^+^). The -NH_3_^+^ exerted repulsive forces to the approaching NH_4_^+^-N that hindered adsorption of the approaching ions onto the CTS-ZMS surface [37]. In an alkaline environment, the OH^−^ and the ammonium nitrogen formed NH_3_, about 10% of NH_4_^+^ was converted to NH_3_ at basic pH [38]. Therefore, the breakthrough time and adsorption capacity decreased at the pH value of 8.5.

#### 3.2.4. Effect of Initial Ammonium Nitrogen Concentration

The effect of initial ammonium nitrogen concentration on the breakthrough curves for a flow rate 32 mL/min, pH value 6.5 and bed depth of 70 cm was shown in Figure 3d. The results showed that increasing ammonium nitrogen concentration (3, 5 and 7mg/L) caused an increase in the adsorption capacity and breakthrough time. *q*_0_ at initial ammonium nitrogen concentration of 3, 5 and 7 mg/L was about 0.095, 0.255 and 0.489 mg/g (Table 3), respectively. This result can be attributed to the greater driving force provided by high concentration of ammonium nitrogen in water for the transfer process to overcome the mass transfer resistance in the filter column [39]. However, the difference between breakthrough time and exhaustion time Δ*t* was shorter than other two processes, which indicated high influent concentration led to shorter running time of the column.

### 3.3. Dynamic Adsorption Model

In this study, the Thomas model was employed to evaluate the adsorption performance of ammonium nitrogen in the filter column of CTS-ZMS. As a dynamic analytical method, the Thomas model is one of the most widely used in the filter column performance [40]. It can provide important system parameters (breakthrough time, adsorption capacity, etc.) which can be used to predict some useful parameters for large scale application [41]. The model parameters like *K_Th_* and *q*_0_ were obtained by using curve fitting approach, the results and the experimental condition were shown in Table 3. 

The high values of the correlation regression coefficient (*R^2^* > 0.90) calculated for the tested parameters (No.1–No.12) indicated that the Thomas model can satisfactorily describe the dynamic adsorption of ammonium nitrogen in the filter column of CTS-ZMS. As seen from Table 3, column adsorption capacity *q_0_* increased with bed depth increasing (No.1–No.3), while for the same bed depth (70 cm), a slight decrease of *q_0_* was observed with flow rate increasing (No.4–No.6). As mentioned in the previous section, increasing concentration of ammonium nitrogen increased the concentration gradient in the filter column, then created a higher driving force and higher number of the adsorption sites, thereby the adsorption capacity increased at higher influent concentrations [42]. The value of *K_Th_* decreasing (No.10–No.12) was due to the fact that the higher concentration of ammonium nitrogen decelerated the rate of mass transfer by lengthening the contact time between the solution and CTS-ZMS adsorbent [43]. Increasing of the flow rate caused *K_Th_* to increase and *q*_0_ to decrease (No.7–No.9). That was because increasing the flow rate decreased the contact time of ammonium nitrogen in the filter column.

### 3.4. SEM 

**Figure 4:**
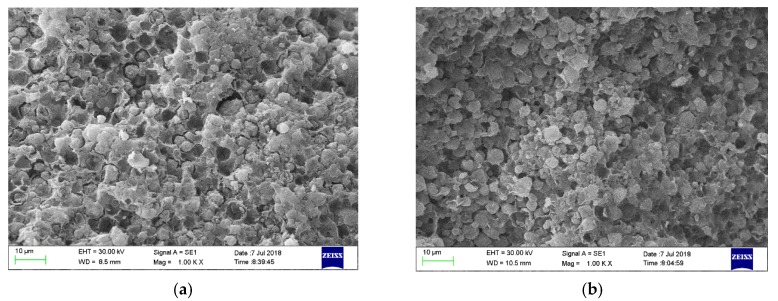
SEM image of the CTS-ZMS samples before and after ammonium nitrogen adsorption: (**a**) CTS-ZMS (×1000), (**b**) ammonium nitrogen adsorbed CTS-ZMS (×1000).

Figure 4a,b illustrated the surface morphology of new CTS-ZMS adsorbent and after running 3 months adsorbed with ammonium nitrogen from the filter column at 1000 magnification, respectively. The surface of CTS-ZMS adsorbent was composed of cubic crystals with regular morphology, which conformed to the morphology of the zeolite NaA type [37]. According to the detection result of BET, the surface area, the average pore diameter and pore volume of CTS-ZMS were 20.782 m^2^/g, 3.56 nm and 0.097 cm^3^/g, respectively. The chitosan was loaded on the surface of the zeolite molecular sieve, therefore the CTS-ZMS surface was formed with more concavo-convex structure and the larger pores. After running three months, there were no obvious changes in the morphology of the CTS-ZMS.

### 3.5. EDS

**Figure 5:**
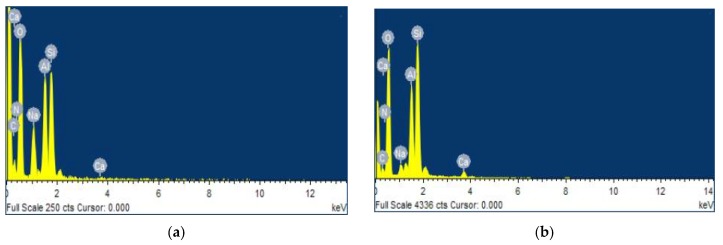
Energy dispersive X-ray spectroscopy (EDS) of the CTS-ZMS samples before and after ammonium nitrogen adsorption: (**a**) CTS-ZMS; (**b**) ammonium nitrogen adsorbed CTS-ZMS.

Figure 5 and Table 4 showed the composition of CTS-ZMS adsorbent before and after 3 months running in a filter column evaluated by EDS analysis. 

As Table 4 showed, O (46.77%), Si (22.35%), Al (10.48%) were the main compensation elements in both CTS-ZMS adsorbent before and after 3 months running. It showed that the aluminosilicate structure framework of zeolite molecular sieve was not changed. After adsorption with ammonium nitrogen, the weight percentage of N (5.16%) element increased to 10.25% on CTS-ZMS, which indicated the CTS-ZMS reacted with ammonium. The content of Na element decreased after adsorption, which showed parts of Na^+^ exchanged with NH_4_^+^ [44].

### 3.6. FTIR

**Figure 6:**
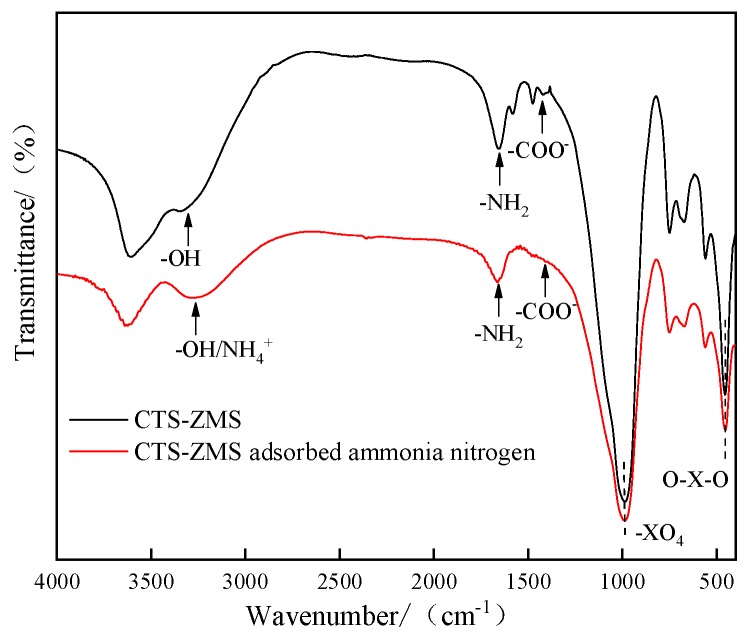
FTIR spectra of the CTS-ZMS samples before and after ammonium nitrogen adsorption.

FTIR spectra from the scanning range 4000 to 400 cm^−1^ of CTS-ZMS adsorbent before and after ammonium nitrogen adsorption were shown in Figure 6. The FTIR spectra of the CTS-ZMS (red line and black line) illustrated the band at 1654 cm^−1^ corresponded to the -NH_2_ bond of the amine group while the band at 1418 cm^−1^ was attributed to -COO^−^ bond, these two bands indicated the typical group of chitosan [45]. The strong bands at 992 cm^−1^ and 453 cm^−1^ were caused by –XO_4_ and O–X–O groups, respectively (X means Si/Al), and the band at 3332 cm^−1^ corresponded to the Si-OH group and Al-OH group on the surface of zeolite molecular sieve framework [46]. The results showed that the chitosan had been successfully loaded onto the surface of zeolite molecular sieve.

A significant change in the intensity of peak was observed for -OH group after ammonium nitrogen adsorption, the position of the peak was shifted from 3332.82 cm^−1^ to 3270.09 cm^−1^, and the position of -NH_2_ group peak was shifted from 1654cm^−1^ to 1669cm^−1^, which indicated the -OH and -NH_2_ had important effects on the adsorption of ammonium nitrogen. The absorption intensities of the –COO^−^ group at 1418 cm^−1^ decreased after ammonium nitrogen adsorption (red line), which indicated that the group of –COO^−^ was related to ammonium nitrogen adsorption reaction. The 3271 cm^−1^ band corresponding to the bond of NH_4_^+^ appeared in the spectrum of CTS-ZMS after adsorption [41]. The bands of -XO_4_ and O-X-O groups did not change before and after the adsorption of NH_4_^+^-N, indicating that the adsorption of NH_4_^+^-N did not change the basic structure of the CTS-ZMS adsorbent.

### 3.7. Adsorption Mechanism

**Figure 7:**
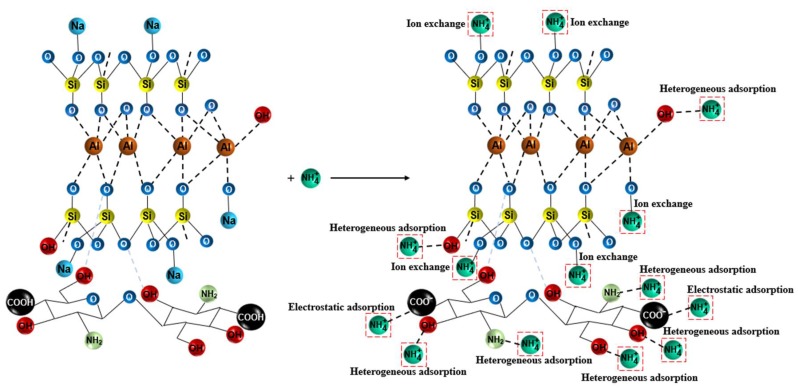
Adsorption mechanism of CTS-ZMS for ammonium nitrogen adsorption.

The possible adsorption mechanism of ammonium nitrogen onto the CTS-ZMS surface can be explained from different perspectives. Characterization tests (EDS and FTIR) were carried out to understand the element and functional groups (-OH, -NH_2_ and -COO^−^) present on the surface of CTS-ZMS adsorbents before and after use. From the EDS analysis, the weight percentage of Na ions decreased and N ions increased after reaction, which indicated ion exchange occurred at the pores of the adsorbent and its internal surface [47,48]. The zeolite molecular sieve framework contained Na ions, therefore this may confirm that the ion exchange was the main mechanism of ammonium ions removal by ZMS [49]. Al-Ghouti et al. (2005) provided evidence that the adsorption of ammonium nitrogen onto zeolite was mainly a chemical reaction rather than physisorption, and the adsorption process can be related to ion-exchange reaction [50], as shown in Figure 7. According to the FTIR analysis, the intensity of peaks for -OH group, -NH_2_ group, -COO^−^ group were changed after ammonium nitrogen adsorption, indicated that the active functional groups -OH, -NH_2_ and -COO^−^ reacted with ammonia nitrogen [51]. Zheng et al. (2009) investigated the adsorption process between ammonium nitrogen and -COO^−^ group was mainly controlled by the electrostatic attraction [52]. Luna et al. (2018) illustrated hydroxyl (-OH) and amine groups (-NH_2_) of CTS were involved in the heterogeneous adsorption mechanism of NH_4_^+^-N [38].

### 3.8. Regeneration

**Figure 8:**
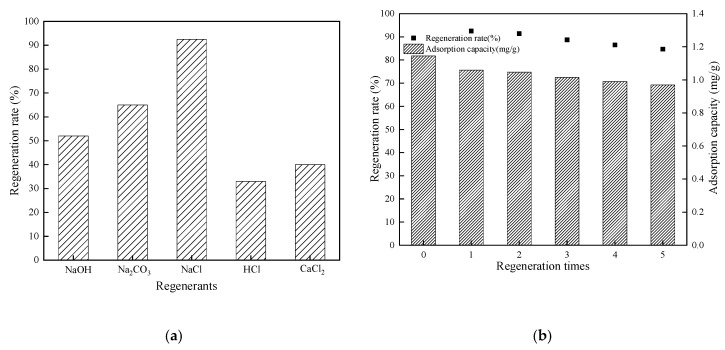
Regeneration results of CTS-ZMS (**a**) regenerants and (**b**) regeneration times.

The regeneration rates of the CTS-ZMS absorbents by NaOH, Na_2_CO_3_, HCl, CaCl_2_ and NaCl were 52%, 65%, 33%, 40% and 92.5%, respectively (Figure 8a), which indicated NaCl solution was the most efficient regenerant. Figure 8b showed the regeneration rates of CTS-ZMS by NaCl after different regeneration times. The initial adsorption capacity of CTS-ZMS was 1.145 mg/g. After 5 times regeneration, the adsorption capacity of CTS-ZMS was 0.97 mg/g and regeneration rate was 84.7% (Figure 8b). This proved that NaCl had a good regeneration effect on CTS-ZMS, and it still had good adsorption performance after 5 regenerations.

## 4. Conclusions

Few literatures reported the chitosan modified zeolite molecular sieve adsorbent (CTS-ZMS) on the dynamic adsorption of ammonium nitrogen. The results of this study demonstrated the effectiveness of the CTS-ZMS adsorbent for the removal of ammonium nitrogen from water via filter column. The adsorption capacity of NH_4_^+^-N through a filter column was dependent on bed depth, flow rate, initial pH value and influent ammonium nitrogen concentration. The Thomas model was found to predict the design parameters successfully. EDS and FTIR analysis revealed that Na ions on the zeolite molecular sieve, -COO^−^ group, -OH group and -NH_2_ group on the chitosan had participated the reaction with ammonium nitrogen. The ion exchange (Na^+^), the electrostatic adsorption (-COO^−^ group) and heterogeneous adsorption (-NH_2_ group) were involved in the reaction with ammonium nitrogen. Dynamic application of CTS-ZMS adsorbents investigated in this study showed efficiency removal of ammonium nitrogen using a filter column. Hence, CTS-ZMS adsorbents can be expected to be a feasible adsorption treatment method for the ammonium nitrogen polluted drinking water.

## Figures and Tables

**Table 1:** The isotherm adsorption model parameters.

	Langmuir			Freundlich		
Type	*q*_m_/(mg/g)	*b/(L/mg)*	*R* ^2^	*K*_f_/[(mg/g)/(mg/L)^1/*n*^]	1/*n*	*R* ^2^
CTS-ZMS	1.145	0.703	0.9504	0.224	0.137	0.6314

**Table 2:** A comparative evaluation of adsorption capacity of other experimental conditions of different adsorbents for NH_4_^+^-N removal.

Adsorbent	Experimental Conditions	NH_4_^+^-N Removal Efficiency	References
CTS	pH: 7.5 Concentration: 22.91 mg/L	29.74%	[18]
Iranian natural zeolite	pH: 7.0 Concentration: 50 mg/L Temperature: 20 °C	69%	[34]
CTS-ZMS	pH: 7.0 Concentration: 4–5 mg/L Temperature: 25 °C	0.636 mg/g 81.60%	[27]
NaA zeolite/chitosan	Adsorbent dose: 0.7 g/L Concentrationrage: 100 mg/L Temperature: 25 °C	5.84 mg/g 82.80%	[35]

**Table 3:** Experimental parameters of the ammonium nitrogen adsorptive removal in the fixed-bed column packed with CTS-ZMS.

Experimental Conditions								Thomas Parameters		
No.	*Z* (cm)	*Q* (mL/min)	pH	*C_0_* (mg/L)	*t_a_* (min)	*t_b_* (min)	Δ*t* (min)	*K_Th_* × 10^−3^ (L/min·mg)	*R* ^2^	*q*_0_/(mg/g)
1	30	32	6.5	5	6	391	385	1.88	0.931	0.225
2	50	32	6.5	5	9	509	500	1.53	0.962	0.228
3	70	32	6.5	5	45	687	642	1.32	0.988	0.255
4	70	32	6.5	5	45	687	642	1.32	0.989	0.255
5	70	49	6.5	5	12	389	377	1.94	0.913	0.182
6	70	65	6.5	5	7	215	208	3.59	0.958	0.123
7	70	32	4.5	5	19	539	520	1.57	0.979	0.215
8	70	32	6.5	5	45	687	642	1.32	0.989	0.255
9	70	32	8.5	5	9	471	462	1.62	0.958	0.190
10	70	32	6.5	3	8	450	442	3.58	0.991	0.095
11	70	32	6.5	5	45	687	642	1.32	0.989	0.255
12	70	32	6.5	7	215	746	531	1.30	0.964	0.489

Note: *Z* = bed depth, *Q*=flow rate, *C_0_* = influent ammonium nitrogen concentration, *t_a_* = breakthrough time, *t_b_* = exhaustion time, Δ*t* = *t_b_ − t_a,_ K_Th_* = Thomas rate constant, *R^2^* = correlation regression coefficient, *q*_0_ = adsorption capacity derived from the Thomas model.

**Table 4:** Element percentage of CTS-ZMS before and after ammonium nitrogen adsorption in fixed-bed column.

	Element (wt.%)	C	N	O	Na	Al	Si	Ca	Total Amount
CTS-ZMS	Weight percentage	10.33	5.16	46.77	4.68	10.48	22.35	0.23	100.00
	Atomic percentage	14.99	6.55	52.40	3.51	6.69	13.35	0.10	
NH_4_^+^-N adsorbed CTS-ZMS	Weight percentage	3.26	10.25	50.22	1.20	13.34	21.43	0.30	100.00
	Atomic percentage	5.15	13.67	57.45	0.94	8.89	13.77	0.13	
